# Direct and Intestinal Epithelial Cell-Mediated Effects of TLR8 Triggering on Human Dendritic Cells, CD14^+^CD16^+^ Monocytes and γδ T Lymphocytes

**DOI:** 10.3389/fimmu.2017.01813

**Published:** 2017-12-22

**Authors:** Costanza Angelini, Barbara Varano, Patrizia Puddu, Maurizio Fiori, Antonella Baldassarre, Andrea Masotti, Sandra Gessani, Lucia Conti

**Affiliations:** ^1^Department of Hematology, Oncology and Molecular Medicine, Istituto Superiore di Sanità, Rome, Italy; ^2^Center for Gender-Specific Medicine, Istituto Superiore di Sanità, Rome, Italy; ^3^Department of Food Safety, Nutrition and Veterinary Public Health, Istituto Superiore di Sanità, Rome, Italy; ^4^Bambino Gesù Children’s Hospital-IRCCS, Research Laboratories, Rome, Italy

**Keywords:** cell activation, pathogen recognition, inflammation, microenvironment, adjuvant

## Abstract

Toll-like receptor (TLR)7/8 plays a crucial role in host recognition/response to viruses and its mucosal expression directly correlates with intestinal inflammation. The aim of this study was to investigate the role of TLR7/8 stimulation of intestinal epithelium in shaping the phenotype and functions of innate immunity cell subsets, and to define direct and/or epithelial cell-mediated mechanisms of the TLR7/8 agonist R848 immunomodulatory activity. We describe novel, TLR8-mediated, pro- and anti-inflammatory effects of R848 on *ex vivo* cultured human blood monocytes and γδ T lymphocytes, either induced by direct immune cell stimulation or mediated by intestinal epithelial cells (IEC). Apical stimulation with R848 led to its transport across normal polarized epithelial cell monolayer and resulted in the inhibition of monocyte differentiation toward immunostimulatory dendritic cells and Th1 type response. Furthermore, γδ T lymphocyte activation was promoted following direct exposure of these cells to the agonist. Conversely, a selective enrichment of the CD14^+^CD16^+^ monocyte subpopulation was observed, which required a CCL2-mediated inflammatory response of normal epithelial cells to R848. Of note, a TLR-mediated activation of control γδ T lymphocytes was promoted by inflamed intestinal epithelium from active Crohn’s disease patients. This study unravels a novel regulatory mechanism linking the activation of the TLR8 pathway in IEC to the monocyte-mediated inflammatory response, and highlights the capacity of the TLR7/8 agonist R848 to directly enhance the activation of γδ T lymphocytes. Overall these results expand the range of cell targets and immune responses controlled by TLR8 triggering that may contribute to the antiviral response, to chronic inflammation, as well as to the adjuvant activity of TLR8 agonists, highlighting the role of intestinal epithelium microenvironment in shaping TLR agonist-induced responses.

## Introduction

Toll-like receptors (TLR) play a fundamental role in pathogen recognition by immune cells leading to immune response activation and pathogen clearance (1). They also control intestinal homeostasis by sensing commensal microorganisms and avoiding detrimental responses.

TLR7 and TLR8 are expressed by myeloid cells, lymphocytes and intestinal epithelial cells (IEC) (2) and recognize specific moieties in viral ssRNA. Their triggering results in viral antigen presentation and generation of protective immune response (3). Conversely, their altered expression in epithelial and lamina propria immune cells may contribute to chronic intestinal inflammation. In particular, TLR7/8 expression in colonic mucosa is increased following antibiotic-induced dysbiosis in mice (4), and abnormal TLR8 expression/signaling characterizes chronic intestinal inflammation contributing to the pathogenesis of inflammatory bowel diseases (IBD) and to inflammation-associated tumorigenesis (5). This receptor is selectively activated in inflamed colonic epithelium of IBD subjects (6, 7) and its expression directly correlates with the severity of intestinal inflammation (8). Furthermore, TLR8 expression characterizes gastrointestinal tumors and correlates with metastasis and poor prognosis (9).

The evidence that different, even opposite, effects can be elicited by TLR7/8 stimulation highlights the importance of a deeper characterization of cell types and immune functions that can be targeted when TLR7/8 agonists are used as vaccine adjuvants. In this regard, the TLR7/8 agonist R848 (also known as resiquimod, S-28463), has come to light as an effective mean of enhancing both antiviral and antitumor responses. R848 is a hydrophobic, low molecular weight synthetic compound, belonging to the imidazoquinoline family and recognizing both TLR7 and TLR8 (10). This agonist has a demonstrated potential as vaccine adjuvant and cancer therapeutic by virtue of its capacity to induce immune mediators and to directly activate dendritic cells (DC), thus preferentially triggering Th1 type responses and enhancing both humoral and cellular immunity (11, 12). However, despite its strong adjuvant properties in mice following topic and oral administration (13–15), the use of R848 in humans has been hampered by their capacity, not yet understood, to rapidly reach the bloodstream and to exert strong systemic effects, mostly dependent on cytokine storm induction (16). Nevertheless, whether and how this compound is adsorbed and/or diffuses across epithelia and tissues, and the role of the delivery route and tissue microenvironment in shaping TLR agonist-induced responses have been only poorly investigated.

The aim of this study was to identify novel cell types and/or immune functions controlled by TLR7/8 triggering of the intestinal epithelium, and to define direct and/or epithelial cell-mediated mechanisms of the R848 immunomodulatory activity. We report that R848 easily diffuses across the polarized Caco-2 cell monolayer and, through TLR8 triggering, it delivers both inflammatory and regulatory signals to monocytes and γδ T lymphocytes, either directly or through epithelial cell stimulation. Specifically, this agonist, when transported across normal epithelial cell monolayer, directly impairs the differentiation of monocytes toward immunostimulatory DC and promotes the activation of γδ T lymphocytes. Simultaneously, it drives the enrichment of the CD14^+^CD16^+^ monocyte subpopulation by stimulating inflammatory chemokine production by IEC.

These results unravel a novel regulatory mechanism linking the activation of the TLR8 pathway in IEC to the monocyte-mediated inflammatory response, and highlight the capacity of R848 to directly enhance the activation of γδ T lymphocytes. This expands the range of cell targets and immune cell responses controlled by TLR8 triggering that may contribute to the antiviral response, to chronic inflammation, as well as to the adjuvant activity of TLR8 agonists.

## Materials and Methods

### Culture of Polarized Caco-2 Cells

Polarized IEC monolayer was obtained by culturing Caco-2 cells (ATCC #HTB-37, 8 × 10^4^ cell/cm^2^) on polycarbonate-coated trans-well chambers (0,4 µm pore, 24 mm diameter, Corning) in high glucose DMEM plus 10% FBS and non-essential amino acids for 21 days. Medium was changed every 2 days and IEC differentiation and integrity was monitored by measuring transepithelial electrical resistance (TEER) throughout the culture period by a Volt-meter (Millicell ERS; Millipore Co., Bedford, MA, USA). At the end of the differentiation period (TEER values >800 Ω cm^2^), medium was changed, DMEM was replaced by RPMI plus 10% FBS at the basolateral side (BS), and cultures were stimulated at the apical side (AS) with R848 (Resiquimod, 5 µg/ml, kindly provided by Dr. Philippe Neuner, IRBM), CL264 (5 µg/ml, InvivoGen) or β-glucan (10 µg/ml, 1 → 3 β-glucan from baker’s yeast, Sigma Aldrich) for 24 h. Agonist concentration 5-fold higher with respect to that used for immune cells was chosen to stimulate epithelial cells. Conditioned medium (CM) was then collected from the BS, filtered and stored at -80°C. IEC monolayer integrity was monitored before and at different time points after treatment by measuring TEER and phenol red transport (17). TEER variations < 10% and phenol red transport ≤ 2% were considered acceptable. Six different preparations of Caco-2 trans-well cultures, leading to comparable results, were used for the study.

### HPLC Analysis of R848 Transport across Intestinal Epithelium

R848 was loaded to the IEC monolayer AS and, at different time points, CM from the BS were collected and analyzed by HPLC for agonist content. HPLC analyses were performed on HPLC system 1100 series coupled to a Diode Array Detector and autosampler (Agilent Technologies, Rome, Italy). The column was a Symmetry C18 reversed-phase (150 mm × 3.0 mm, 5 µm), connected to a Sentry Guard Column Symmetry C18 5 µm (3.9 mm × 20 mm) (Waters, Milford, MA, USA). The mobile phase was constituted by solvent A (water containing glacial acetic acid 1% v/v) and solvent B (acetonitrile). Chromatography was carried out by linear gradient at room temperature, according to the following program: 2% B for 2 min; from 2 to 100% B in 8 min, holding on for 2 min, finally to 2% B in 2 min; the equilibrium time between analyses was 5 min. The flow rate was 0.300 ml/min and 10 µl of sample was injected onto the column. Wavelength λ = 245 nm. The extent of agonist transport was calculated by dividing the cumulative amount of molecule transported, at the different time points, into the BS CM with the original loading concentration. Caco-2 IEC monolayer was also exposed to different concentrations of R848 for 5 h, BS CM was analyzed by HPLC and the apparent permeability coefficients (Papp) were calculated as previously described (18).

### Monocyte-Derived DC Generation and Culture

Monocytes were isolated from the peripheral blood of healthy donors by Ficoll/Paque density gradient centrifugation followed by immunomagnetic selection using CD14^+^ microbeads (MACS monocyte isolation kit, Miltenyi Biotec, Auburn, CA, USA), according to the manufacturer’s instructions. To obtain control immature monocyte-derived DC, monocytes were seeded at 1 × 10^6^ cells/ml in standard medium [RPMI 1640 medium containing 10% FBS, GM-CSF (50 ng/ml, kindly provided by Schering-Plough, Dardilly, France) and IL-4 (500 U/ml, Miltenyi Biotec, Auburn, CA, USA)] and cultured for 5 days. Fresh medium plus cytokines was added at day 3 of culture. For some experiments, monocytes were cultured in standard medium and exposed to R848 (1 µg/ml), CL264 (1–2 µg/ml), or β-glucan (2 µg/ml) soon after seeding. CM-conditioned DC was generated in the same conditions by replacing standard medium with Caco-2 CM. For cytokine blocking, monocytes were seeded in CM after a 30-min pre-incubation of this latter with neutralizing Ab (2.5 µg/ml) to CCL2 (rabbit polyclonal, Sigma Aldrich) or IL-6 (mouse monoclonal, Sigma Aldrich), or with isotype control Ab (rabbit and mouse IgG, respectively). TLR blocking was performed by treating R848 CM-exposed monocytes with phosphorothioate oligonucleotides (ODN) targeting TLR7/8/9 (# 2088, TCCTGGCGGGGAAGT; 1 µM) or specific for TLR7 (# 20958, TCCTAACAAAAAAAT; 2.5 µM) soon after seeding.

### γδ T Lymphocyte Isolation, Culture, and Interaction with DC

γδ T lymphocytes were isolated from cryopreserved PBMC of healthy donors by positive selection with immunomagnetic beads (Miltenyi Biotec), according to the manufacturer’s instructions. Positively selected population contained >95% viable γδ T cells as assessed by flow cytometry. After an overnight culture in complete medium (RPMI plus 10% FBS), purified γδ T cells were washed, suspended in the same medium at the density of 10^6^ cells/ml and exposed to R848 or CL264, or seeded, at the same density, in Caco-2 or primary IEC-derived CM. Cells were stimulated with the non-peptide phosphoantigen isopentenilpyrophosphate (IPP, 2 µg/ml, Sigma Aldrich) and cultured for 48 h.

Dendritic cell/γδ T cell co-cultures were set up by adding purified lymphocytes to autologous control or CM-generated DC (1:1 ratio) as previously described (19). Co-cultures were left untreated or stimulated with the aminobiphosphonate zoledronate (ZOL, 10 µg/ml, kindly provided by Novartis Pharma, Origgio, VA) and analyzed 48 h later.

### CD4^+^ T Cell Activation Assay

Naive CD4^+^ T lymphocytes were isolated from PBMC of healthy donors by negative selection with immunomagnetic beads (Miltenyi Biotec), according to the manufacturer’s instructions. The purity of isolated cells (≥98%) was checked by flow cytometry after labeling with anti-CD4-PE and anti-CD45 RA-FITC.

For T cell activation assay, 5-day cultured control or CM-generated DC were stimulated with LPS (10 ng/ml) for 24 h to obtain mature cells. Mature DC were washed twice in serum-free RPMI and co-cultured with freshly isolated allogeneic naive CD4^+^ T lymphocytes (1:10 ratio), in a mixed leukocyte reaction assay, for 12 days in RPMI plus 5% human AB serum. IL-2 (50 U/ml) was added to the co-cultures at day 6. Lymphocytes were then harvested, counted, seeded in fresh medium and stimulated with PMA (50 ng/ml) and Ionomycin (1 µg/ml). 18 h later, culture medium was collected for IFNγ content determination.

### Ethics Statement

Healthy donor buffy coats were obtained from Centro Trasfusionale, Sapienza University of Rome. Buffy coats were not obtained specifically for this study. Informed consent was asked to blood donors according to the Italian law n. 219 (October 21, 2005), recently revised (D.L. of the Ministry of Health, November 2, 2015). Data have been treated by Centro Trasfusionale according to the Italian law on personal data management “Codice in materia di protezione dei dati personali” (Testo unico D.L. June 30, 2003 n. 196).

Investigation including IBD patients has been conducted in accordance with the Declaration of Helsinki and, according to national and international guidelines. The study was approved by the review board of Istituto Superiore di Sanità (project identification code: CE/11/299). All the subjects included were provided with complete information about the study and asked to sign an informed consent.

### Patients and Biological Samples

Endoscopic biopsies, taken from colon/ileum tissue, were obtained from patients with documented Crohn’s disease (CD, *n* = 8) or age and sex matched healthy controls (HC, *n* = 8, undergoing screening colonoscopy), attending to the Digestive Endoscopy Unit (Catholic University, Rome, Italy). The exclusion criteria were: clinical evidence of active infection, recent (within 14 days) use of antibiotics, pregnancy, hormone-based therapy, and treatment with corticosteroids. Endoscopic activity was assessed according to the Crohn’s Disease Endoscopic Index of Severity (remission when score <3) (20). Biopsies were kept in ice-cold PBS containing penicillin, streptomycin (50 IU/ml) and gentamycin (0.5 mg/ml) (Transport Medium, TM) before enterocyte isolation.

### Isolation of Primary IEC

IEC were isolated from whole biopsies as previously described (21). Briefly, biopsies were extensively washed by shaking in TM, and then incubated in TM containing EDTA and EGTA (1 mM) for 75 min at 21°C under soft stirring. Intestinal crypts were then allowed to detach from mucosa by vigorous shaking and cultured ON in RPMI plus 10% FBS. CM was collected, filtered, and stored at −80°C.

### Determination of Soluble Immune Mediators

Caco-2 CM as well as supernatants from control and CM-generated DC, γδ T lymphocytes, and DC-T cell co-cultures were analyzed by ELISA for their content of CCL2, IL-10, PGE_2_ (R&D Systems Inc., Minneapolis, MN, USA), IL-1β, IL-6, IL-12, TNFα, and IFNγ (Biolegend, San Diego, CA, USA), according to the manufacturer’s instructions.

### Flow Cytometry Analysis

The expression of surface markers was analyzed by flow cytometry. Briefly, cells were pre-incubated for 30 min on ice with PBS containing 10% human AB serum to block non-specific Ig binding and then incubated with the specific Abs or control isotypes for 30 min on ice, washed, and analyzed. The following Abs were used: FITC- or APC-conjugated CD14 (BD Pharmigen or eBiosciences, respectively), PE-CD1a, PE-CD206/MR, PE-CD209/DC-SIGN (BD Pharmigen), and FITC-CD16 (eBiosciences). At least 10,000 events/sample were acquired by a FACScan cytometer (BD Biosciences). Data analysis was performed by gating on the monocyte/DC populations and excluding death cells and debris.

### Immunoblotting Analysis

Whole cell extracts were prepared by lysing cells in RIPA buffer [150 mM NaCl, 50 mM Tris–Cl (pH 7.5), 1% Nonidet P-40, 0.5% sodium deoxycholate, and 0.1% sodium dodecyl sulfate (SDS)] containing a cocktail of protease (Roche) and phosphatase inhibitors (Sigma-Aldrich). Cell lysates (10 µg per lane) were fractionated on 8% SDS-PAGE, transferred to a nitrocellulose membrane, and subjected to immunoblot analysis using antibodies specific for the total or tyrosine phosphorylated forms of STAT3 (Cell Signaling Technology). Equal loading of proteins was verified by blotting the same gel with antibodies to β-actin (BD Biosciences).

### Real-time PCR

Total RNA was isolated from 21-day cultured polarized Caco-2 cell monolayer or freshly isolated blood monocytes and γδ T lymphocytes with Total RNA Purification Plus Kit (Norgen Biotek, Canada). RNA quality was checked on an Agilent 2100 Bioanalyzer and reverse transcribed (SuperScript™ III, ThermoFisher). Glucuronidase beta (GUSB) was used as reference gene. Primers for TLR7 (Hs.PT.58.38778009), TLR8 (Hs.PT.58.15023918.G), and GUSB (Hs.PT.39a.22214857) were purchased from Integrated DNA technologies. mRNA levels were quantified by qPCR on a QuantStudio™ 12K Flex OpenArray^®^ (Applied Biosystems) by using the comparative ΔΔ*C*_t_ method.

### Statistical Analysis

GraphPad Prism 5 software was used for statistical analysis. Statistical comparisons were performed by the one-way analysis of variance, with Newman–Keuls *post hoc* test, for multiple groups and by the two-tailed paired Student’s *t*-test for independent samples, as appropriate. Differences were considered significant when *p* values were <0.05.

## Results

### R848-Conditioned IEC Affect the Differentiation of Monocyte-Derived DC and Their Capacity to Stimulate Th1 Type Responses

**Figure 1:**
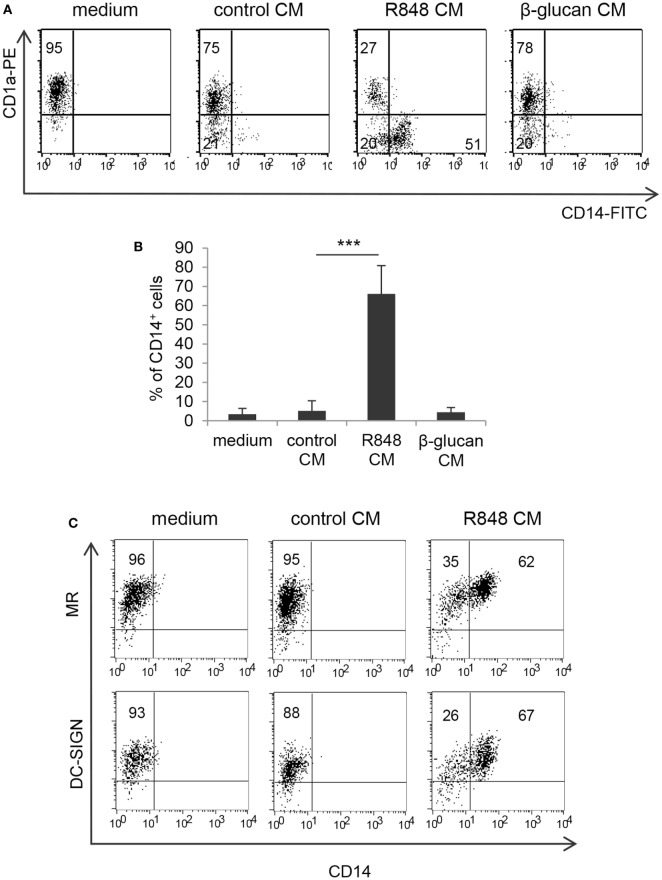
Effects of R848-exposed intestinal epithelial cell (IEC) monolayer on dendritic cell (DC) differentiation. Peripheral blood monocytes were induced to differentiate toward DC in standard medium or in conditioned medium (CM) from Caco-2 cell-derived IEC monolayer, left untreated or stimulated with R848 **(A–C)** or β-glucan **(A,B)**. At day 5, cells were harvested and analyzed for the expression of the indicated surface markers by flow cytometry. One representative experiment out of 4 is reported in panels **(A,C)**. Numbers in quadrants indicate the percentages of positive cells. The percentage of CD14^+^ cells is reported in panel **(B)**, mean values ± SD from 10 independent experiments are shown. ***p < 0.001.

To assess whether TLR7/8 triggering in intestinal epithelium may transduce signals ultimately affecting the functional properties of innate immunity cells, we analyzed the effects of polarized Caco-2 cell monolayer, stimulated with R848, on the differentiation of human monocytes toward DC. Polarized IEC monolayer was left untreated or stimulated, at the AS, with R848. Human peripheral blood monocytes were induced to differentiate toward DC in the presence of control medium or CM from unstimulated or TLR-stimulated Caco-2 cells. As shown in Figures 1A,B, a significant proportion of monocytes exposed to CM from R848-conditioned IEC monolayer (R848 CM) did not express the DC-specific marker CD1a and retained the expression of CD14 as compared to cultures exposed to standard medium, indicative of impaired DC differentiation. Conversely, only a slight reduction in CD1a expression was detected when DC were generated in the presence of control CM (Figures 1A,B). Likewise, DC differentiation was not affected when monocytes were exposed to CM from Caco-2 cells stimulated with β-glucan, an immunomodulatory compound endowed with adjuvant properties, which recognizes a different family of pattern recognition receptor (PRR) (Figures 1A,B).

Despite the negative effect exerted by R848 CM on monocyte to DC differentiation, cells generated under these conditions expressed levels of mannose receptor (MR) and DC-SIGN comparable, or even higher, to those of cells cultured in standard medium (Figure 1C; MR: 98% ± 7.3 vs. 96.7% ± 6.0; DC-SIGN: 97% ± 4.9 vs. 96% ± 6.4; *n* = 4).

**Figure 2:**
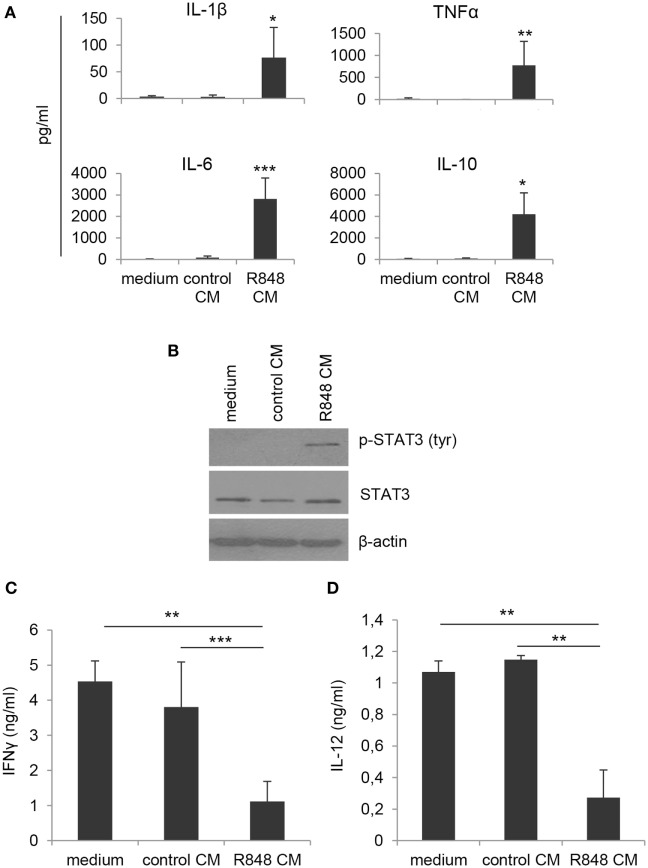
Effects of R848-exposed IEC monolayer on DC functional properties. Monocyte-derived DC were generated as described in the legend to Figure 1 and harvested at day 5 of culture. **(A)** Cell supernatants were analyzed for the release of the indicated cytokines by ELISA. Mean values ± SD from five independent experiments are shown. Comparisons with control CM are reported. **(B)** Western blot analysis of phospho-STAT3 (pSTAT3), total STAT3, and β-actin was performed on protein extracts. A representative blot out of four independent experiments analyzed is shown. **(C)** Cells cultured as in panels **(A,B)** were stimulated with LPS for 24 h and then co-cultured with allogeneic naive CD4^+^ T lymphocytes (1:10 ratio). After 18 h stimulation with PMA and ionomycin, supernatants were collected and analyzed for IFNγ content. Data are shown as mean values ± SD from four independent experiments. **(D)** LPS-stimulated DC cultures were analyzed for IL-12 production by ELISA. Mean values ± SD from three independent experiments are shown. *p < 0.05; **p < 0.01; ***p < 0.001.

By contrast, when DC cultures obtained under the different conditions were analyzed for cytokine expression, significant changes in the profile of cytokines constitutively released were observed. As shown in Figure 2A, cells generated in R848 CM expressed higher levels of both pro-inflammatory (IL-1β, TNFα) and regulatory (IL-6, IL-10) cytokines as compared to cultures generated in control CM and standard medium, suggesting that a heterogeneous population of cells differentiated from R848 CM-stimulated monocytes.

To further investigate the nature of these cells, we analyzed the levels of phosphorylated STAT3, whose activation is induced by both IL-6 and IL-10 and has been reported in cells with regulatory/tolerogenic features. As expected, constitutive STAT3 activation was found in R848 CM-generated cells but not in cells differentiated in standard medium or control CM (Figure 2B). Accordingly, R848 CM-generated cells were found to impair IFNγ production in co-cultured CD4^+^ T lymphocytes (Figure 2C), whereas no effect was observed on the production of IL-17 (data not shown). In line with the effect on Th1 cytokine production, a reduced secretion of the Th1-polarizing cytokine IL-12 was also detected in R848 CM DC-T cell co-cultures (Figure 2D).

### Apical Exposure of Polarized Caco-2 Cells to R848 Results in Agonist Transport across the Epithelial Cell Monolayer

**Figure 3:**
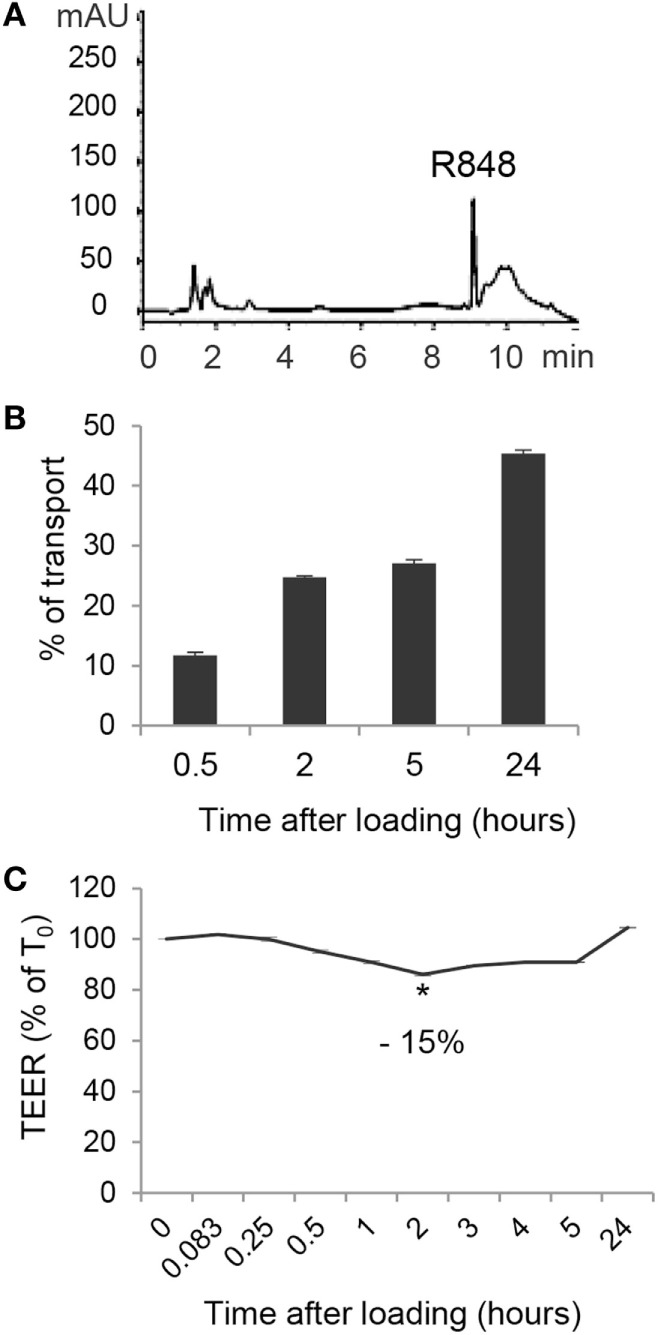
Transport of R848 across intestinal epithelium and effect on epithelium permeability. **(A)** HPLC chromatogram of CM spiked with 5 µg/ml of R848. **(B)** R848 (5 µg/ml) was loaded to the epithelial cell monolayer apical side (AS) and, at the indicated time points, CM from the basolateral side were collected and analyzed by HPLC for agonist content. Total percent agonist transport was calculated by dividing the cumulative amount of molecule transported with the original loading concentrations. Data are expressed as mean values ± SD of triplicate samples and one representative experiment out of three is shown. **(C)** R848 (5 µg/ml) was loaded to the AS and transepithelial electrical resistance (TEER) was monitored before loading (time 0, T0) and at the indicated time points. Data are expressed as percentage of T0. Mean values ± SD of triplicate samples of one representative experiment out of three are shown. The contrast has been adjusted in the entire panel **(A)** to enhance resolution.

Given the highly hydrophobic nature and the small molecular weight of R848, as well as the rapid systemic effects observed in *in vivo* studies following its oral or intracolonic delivery, we therefore investigated whether treatment of polarized Caco-2 cells could result in agonist transport across the monolayer. To this aim, Caco-2 cell monolayer was exposed, at its AS, to R848 and CM from the BS was collected at 0.5, 2, 5, and 24 h and subject to HPLC analysis. A chromatogram of CM spiked with 5 µg/ml of R848 is shown in Figure 3A. A significant proportion of apically loaded R848 was found to be transported to the BS chambers already after 30 min of exposure and this proportion increased overtime, reaching more than 40% of transport at 24 h (Figure 3B). To evaluate whether R848 transport could be somehow related to agonist-induced alteration of epithelial permeability, TEER was monitored before agonist loading and at different time points during treatment. As shown in Figure 3C, a 15% drop in TEER values was observed at 2 h post-treatment, but recovered soon after, suggesting that some reversible R848-induced perturbation of monolayer permeability could also contribute to its transport. Dose-response experiments were then performed in which Caco-2 cell monolayer was apically exposed to different R848 concentrations for 5 h and the apparent permeability was calculated (18, 22). The permeability coefficients obtained (*P*_app_ = 4.52 ± 0.8 × 10^−6^, 3.5 ± 1.7 × 10^−6^, and 3.2 ± 1.8 × 10^−6^ cm/s, with 1, 2, and 5 µg/ml apical loading, respectively) were compatible with complete agonist adsorption.

### R848 CM-Induced Impairment of DC Differentiation/Activation Results from TLR8-Mediated Direct Stimulation of Monocytes by the Transported Agonist

**Figure 4:**
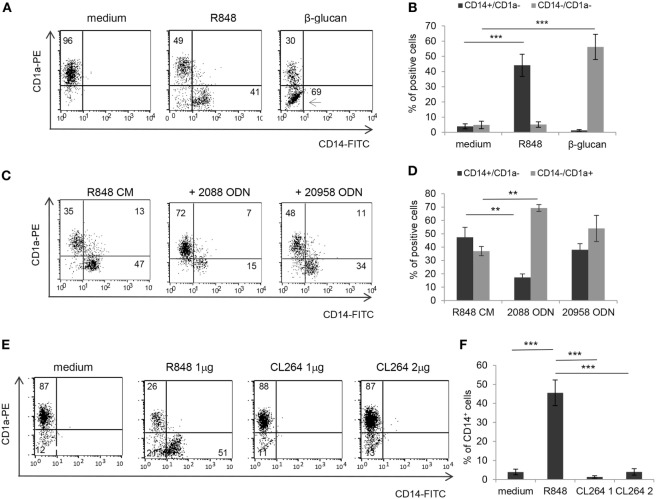
Effects of direct R848 and CL264 stimulation of monocytes on CD14 and CD1a expression and role of TLR8. **(A,B)** Monocytes were induced to differentiate toward DC for 5 days in standard medium in the presence or in the absence of the indicated pattern recognition receptor ligands and analyzed for the expression of CD14 and CD1a by flow cytometry. One representative experiment out of five is shown in panel **(A)**. The percentages of CD14^+^CD1a^−^ and CD14^−^CD1a^−^ cells recovered is reported in panel **(B)**, mean values ± SD from five independent experiments are shown. **(C,D)** Monocytes were exposed to R848 treated Caco-2 CM and induced to differentiate toward DC following pre-incubation with TLR7/8/9 (2088) or TLR7 (20958) targeting ODN. CD14 and CD1a expression was analyzed at day 5 by flow cytometry. One representative experiment out of three is shown in panel **(C)**. The percentages of CD14^+^CD1a^−^ and CD14^−^CD1a^+^ cells obtained is reported in panel **(D)**, mean values ± SD from three independent experiments are shown. **(E,F)** Monocytes were induced to differentiate toward DC for 5 days in standard medium in the presence of R848 (1 µg/ml) or CL264 (1 or 2 µg/ml) and analyzed for CD14 and CD1a expression as in panels **(A–D)**. One representative experiment out of four is shown in panel **(E)**. The percentages of CD14^+^ cells obtained is reported in panel **(F)** as mean values ± SD from four independent experiments. Numbers in quadrants indicate the percentages of positive cells. **p < 0.01; ***p < 0.001.

Based on the finding that R848 is transported through IEC monolayer, we then investigated whether the effects observed with IEC CM could be reproduced after direct stimulation of immune cells by R848 or whether they required epithelial cell response to the agonist. To this aim, the exact concentration of R848 released in the BS CM at the different time points was calculated. A significant effect on the percentage of CD14^+^ cells was only observed when CM from Caco-2 cells exposed for 24 h to 5 µg/ml of R848 was used (1 ± 0.15 µg/ml of agonist released), whereas only barely detectable changes were appreciated when CM derived from cells exposed for shorter times or lower concentrations were used (data not shown). Thus, monocytes were directly exposed to a comparable concentration of R848 (1 µg/ml) and induced to differentiate toward DC. As shown in Figures 4A,B, direct agonist treatment of monocytes resulted in reduced DC differentiation, as demonstrated by the high proportion of CD14^+^CD1a^−^ cells found in these cultures as compared to control cultures. The effect of the direct stimulation with the agonist on DC differentiation was comparable to that of R848 CM (Figure 1A). Furthermore, as observed for R848 CM-generated DC, cells differentiated following direct exposure to R848 upregulated the expression of MR and DC-SIGN and exhibited constitutive cytokine expression (data not shown). Conversely, while β-glucan CM did not exert any effect on DC differentiation (Figure 1A), the exogenous addition of this compound to monocytes resulted in a strong enrichment of CD14/CD1a double negative cells with respect to untreated control cultures (Figures 4A,B), in keeping with previously reported data (23). The biochemical properties of β-glucan might influence its transport through epithelial cells thus providing an explanation for the divergent effect of this compound with respect to R848.

As a further evidence that the effect of R848-conditioned epithelial cells on DC differentiation is dependent on the agonist released in CM, and to investigate the relative contribution of TLR7 and TLR8 to this effect, monocytes were cultured in R848 CM after pre-exposure to phosphorothioate ODN targeting TLR7/8/9 (#2088) or TLR7 (#20958). An almost complete recovery of CD14^−^CD1a^+^ DC was obtained only in the presence of the TLR7/8/9 targeting ODN, whereas the TLR7-specific ODN did not exert any significant effect (Figures 4C,D), thus suggesting a selective role of TLR8. In keeping with these results, monocytes were found to express significantly higher levels of TLR8 with respect to TLR7 (Figure S1 in Supplementary Material). The selective requirement of TLR8 stimulation was also confirmed in experiments in which DC differentiation was induced following monocyte exposure to the TLR7 specific ligand CL264 (Figures 4E,F).

### R848-Conditioned Epithelial Cell Monolayer, but Not Direct R848 Exposure of Monocytes, Drives the Accumulation of the CD14^+^CD16^+^ Subpopulation: Role of Epithelial Cell-Derived CCL2

**Figure 5:**
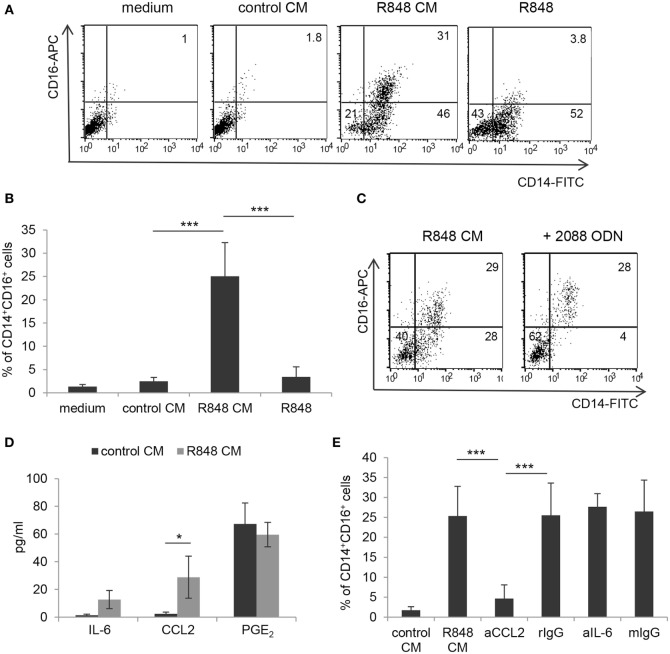
Selective induction of CD14^+^CD16^+^ cell enrichment by R848-conditioned epithelium microenvironment and role of CCL2. **(A,B)** Monocytes were induced to differentiate toward DC by culture either in standard medium, in the presence or in the absence of R848, or in control or R848 Caco-2 CM. At day 5, cultures were analyzed for the expression of the indicated surface markers by flow cytometry. **(C)** Monocytes were cultured in R848 Caco-2 CM following pre-incubation with TLR7/8/9 targeting ODN and analyzed as in panels **(A,B)**. **(D)** Polarized Caco-2 cell monolayer was left untreated or stimulated with R848 for 24 h. CM was collected and assessed for the secretion of the indicated immune mediators. **(E)** Monocytes were exposed for 5 days to control or R848 CM, left untreated or pre-incubated with neutralizing Ab to the indicated cytokines or with isotype control Ab, and analyzed as in panels **(A,C)**. aCCL2/aIL-6 (anti-CCL2 Ab/anti-IL-6 Ab), rIgG (rabbit polyclonal IgG), and mIgG (mouse monoclonal IgG). Numbers in quadrants **(A,C)** indicate the percentages of single and double positive cells. Mean values ± SD from five, five, and four independent experiments are shown in panels **(B,D,E)**, respectively. *p < 0.05; ***p < 0.001.

It has been reported that R848 injection in non-human primates results in a decrease of blood myeloid DC and concomitant enrichment of the CD14^+^CD16^+^ monocyte subpopulation, known to be associated with several inflammatory conditions (24, 25). To investigate whether R848 conditioning of IEC monolayer could affect the monocyte subset distribution, monocyte cultures exposed for 5 days to control or R848 CM were analyzed for the expression of CD14 and CD16. Figures 5A,B show that a proportion of the CD14^+^ cells obtained upon monocyte exposure to R848 CM (see Figure 1A) indeed co-expressed CD16. As expected, cells cultured in control CM or standard medium neither expressed CD14 nor CD16 (Figures 5A,B). A donor-dependent variable percentage (3.1–9.8%) of CD14/CD16 double positive cells was present in the starting population of freshly isolated CD14^+^ monocytes.

To analyze whether direct agonist exposure of monocytes could be responsible for the induction of CD14^+^CD16^+^ cells, parallel monocyte cultures were exogenously stimulated with R848 and checked for the appearance of double positive cells. In contrast to what observed with R848 CM, when monocytes were directly exposed to a comparable (1 µg/ml, Figures 5A,B) or even higher (up to 4 µg/ml, data not shown) concentration of the agonist, the population of CD14/CD16 double positive cells was no longer identified. Experiments with the TLR7/8 targeting ODN further confirmed that R848 CM-induced enrichment of this subset was driven by the epithelium microenvironment and not by direct stimulation of monocytes with the agonist transported across the epithelial cell monolayer (Figure 5C). Specifically, the addition of TLR7/8/9 ODN to monocytes completely reduced the proportion of CD14^+^CD16^−^ cells recovered (Figure 5C; 2.5 ± 1.7 vs. 35 ± 9.5%, *n* = 3, *p* < 0.001) without affecting that of the CD14/CD16 double positive cells (Figure 5C; 25 ± 8.7 vs. 28 ± 7.53%, *n* = 3, *p* < 0.001). No effect was exerted on this latter cell population by the TLR7-specific ODN as well (data not shown).

Notably, when monocytes were exposed to CM from Caco-2 cells stimulated with the TLR7 selective agonist CL264, the CD14^+^CD16^+^ cell enrichment was no longer observed [1.83 ± 0.9% (CL264 CM) vs. 28 ± 7.53% (R848 CM), *n* = 3, *p* < 0.001]. This suggested that the accumulation of this monocyte subpopulation induced by R848 CM requires TLR8 recognition on IEC. Accordingly, TLR8 was found to be expressed at higher levels with respect to TLR7 in Caco-2 cell monolayer (Figure S1 in Supplementary Material).

To the aim of identifying the soluble factor(s) released by R848-stimulated Caco-2 cell monolayer that could be responsible for CD14^+^CD16^+^ cell enrichment, we focused on pro-inflammatory mediators relevant for intestinal inflammation, such as IL-6, CCL2, and PGE_2_. As shown in Figure 5D, the expression of CCL2, but not of IL-6, was significantly induced in R848-stimulated Caco-2 cell cultures as compared to unstimulated cultures. Conversely, comparable levels of PGE_2_ were found in both untreated and R848-exposed epithelial cells (Figure 5D). Based on this evidence, monocytes were then induced to differentiate into DC in the presence of R848 CM previously incubated with neutralizing antibodies to CCL2 or IL-6. The results of this analysis showed that CCL2 but not IL-6 blocking almost completely abolished the appearance of the CD14^+^CD16^+^ cell population (Figure 5E). The possible role of type I IFN was also investigated, but neutralizing Ab to these cytokines failed to affect monocyte phenotype (data not shown).

### Epithelial Cell Microenvironment Impairs DC-γδ T Cell Crosstalk Independently of R848 Conditioning

**Figure 6:**
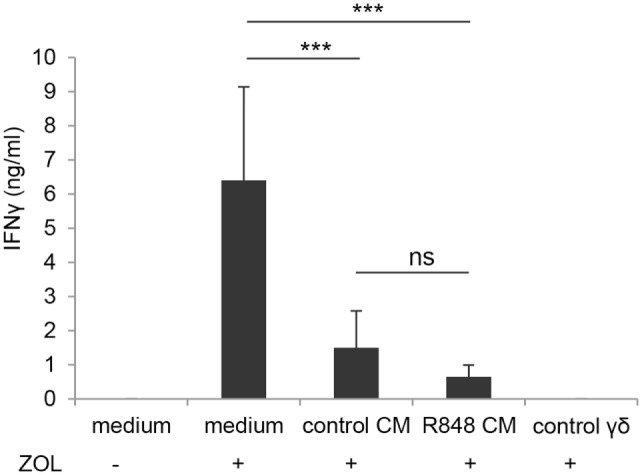
Effect of epithelial cell microenvironment on DC-mediated activation of γδ T lymphocytes. DC generated in standard medium or in control or R848 Caco-2 CM were co-cultured with autologous γδ T lymphocytes (1:1 ratio) for 48 h in the presence (+) or in the absence (−) of zoledronate (ZOL). Co-culture medium was collected and analyzed for IFNγ content. ZOL-stimulated γδ T cells were used as a control. Data are expressed as mean values ± SD from five independent experiments. ***p < 0.001.

In light of the negative effect on CD4^+^ T cell-mediated IFNγ production exerted by DC generated in R848 CM (Figure 2C), we further analyzed the impact of these cells on the activation of γδ T lymphocytes. We previously reported that aminobiphosphonate-induced activation of γδ T cells requires the presence of DC (19). Thus, DC generated in the presence of control or R848 Caco-2 CM were co-cultured with ZOL-stimulated autologous γδ T lymphocytes, and lymphocyte activation was assessed by measuring IFNγ secretion. As shown in Figure 6, DC generated in Caco-2 CM, regardless of R848 stimulation, exhibited a significantly reduced capacity to activate γδ T lymphocytes, as assessed by the lower levels of IFNγ released in the co-culture medium with respect to that of control DC generated in standard medium. This suggests that signals delivered by intestinal epithelium in homeostatic conditions may negatively regulate the DC/γδ T cell cross-talk and that R848 exposure neither potentiates nor counteracts this immunosuppressive microenvironment.

### R848 CM and Direct R848 Exposure Potentiate Phosphoantigen-Induced Activation of γδ T Lymphocytes *via* TLR8

**Figure 7:**
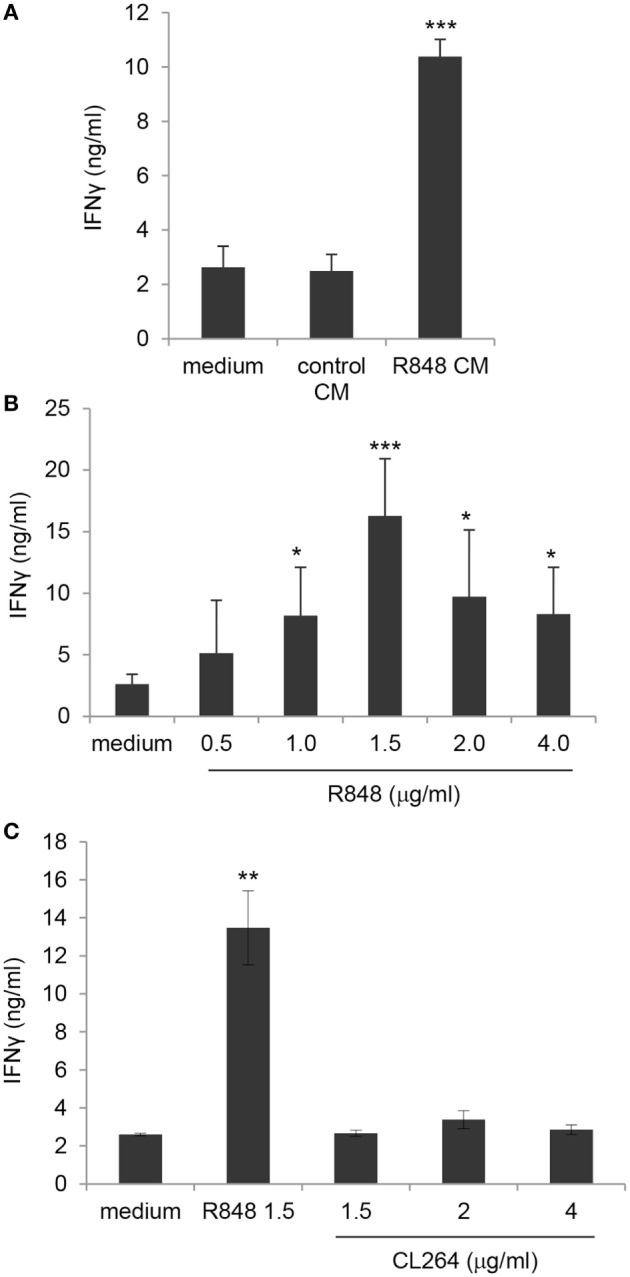
Enhancement of γδ T lymphocyte activation by direct exposure to R848 and role of TLR8. **(A)** Positively selected blood γδ T lymphocytes were stimulated with isopentenilpyrophosphate (IPP) in the presence of standard medium or in control or R848 Caco-2 CM, and analyzed 48 h later for IFNγ production. **(B,C)** γδ T cells were stimulated with IPP in standard medium in the absence or in the presence of the indicated concentrations of R848 or CL264, and analyzed as in panel **(A)**. Data are expressed as mean values ± SD from five **(A,B)** and four **(C)** independent donors. Comparisons with medium and control CM **(A)**, with medium **(B)** and with medium and CL264 **(C)** are reported. *p < 0.05; **p < 0.01; ***p < 0.001.

In order to evaluate the effect of R848-conditioned epithelial cells on the direct, DC-independent activation of γδ T cells, purified γδ T lymphocytes were stimulated with the non-peptide phosphoantigen IPP in the presence of control or R848 CM and analyzed for IFNγ secretion. As shown in Figure 7A, direct γδ T cell activation was not affected by their exposure to CM from unstimulated epithelial cell monolayer as comparable levels of IFNγ were released by cells cultured in control CM and standard medium. In contrast, lymphocytes cultured in R848 CM expressed significantly higher levels of this cytokine (Figure 7A), indicating that agonist stimulation may provide a suitable microenvironment sustaining their activation.

Based on previously reported data (26) as well as on our results (Figure S1 in Supplementary Material), showing that human circulating γδ T lymphocytes express both TLR7 and TLR8, we then assessed whether the effect of R848 CM on γδ T cell activation was due to their direct exposure to the released agonist. Thus, purified blood γδ T cell cultures were kept in standard medium and simultaneously stimulated with IPP and R848. As shown in Figure 7B, the agonist was *per se* able to significantly increase TCR-mediated IFNγ production at concentrations comparable to those found in Caco-2 CM, thus demonstrating that R848 transported across Caco-2 cell monolayer is responsible for γδ T lymphocyte hyper-activation. According to the higher IFNγ release, an increased proportion of CD25-positive cells was observed in γδ T cell cultures exposed to both R848 and R848 CM (data not shown).

To further investigate the relative contribution of TLR7 and TLR8 to the R848-mediated effect on γδ T lymphocytes, these cells were exposed to comparable concentrations of the TLR7 specific ligand CL264. As shown in Figure 7C, stimulation of lymphocytes with the TLR7 agonist did not increase IPP-induced IFNγ production, thus pointing to TLR8 as the main mediator of R848 effect. Accordingly, TLR8 was expressed at higher levels with respect to TLR7 in these cells (Figure S1 in Supplementary Material).

### TLR-Mediated Activation of γδ T Lymphocytes Is Promoted by Inflamed Intestinal Epithelium from Active CD Patients

**Figure 8:**
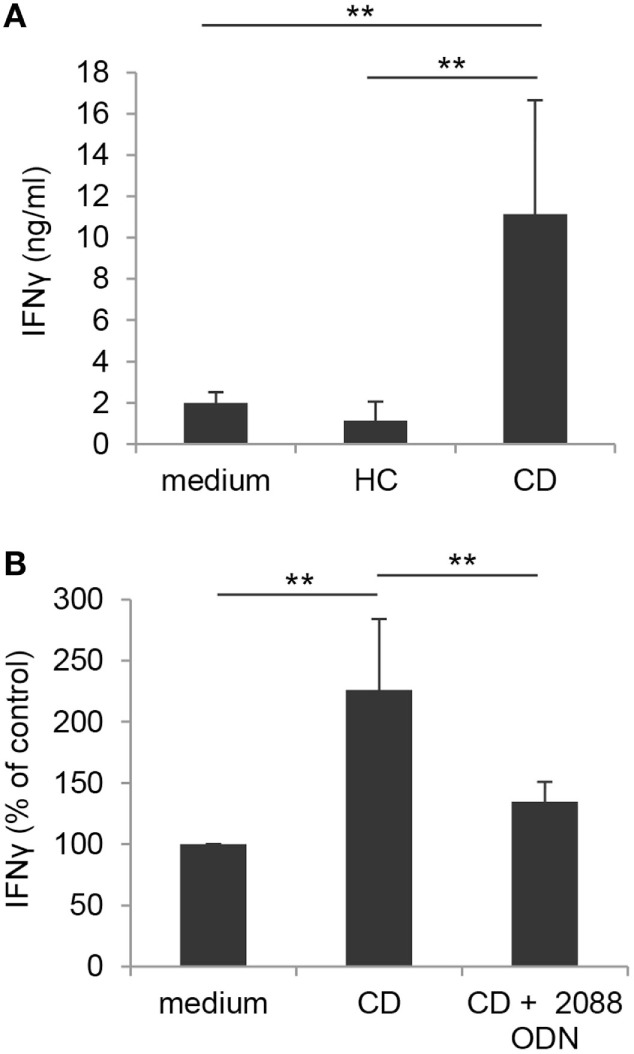
Toll-like receptor-mediated enhancement of γδ T lymphocyte activation by CM of IEC from active Crohn’s disease (CD) patients. **(A)** γδ T lymphocytes, isolated from healthy donors, were exposed to medium or to CM of IEC collected from either active CD patients or HC, stimulated with IPP and analyzed 48 h later for IFNγ production. Mean values ± SD are shown, *n* = 8. **(B)** Control γδ T lymphocytes, isolated as in panel **(A)**, were exposed to CM of IEC from active CD patients, stimulated with IPP in the presence or absence of TLR7/8/9 targeting ODN (2088), and analyzed as in panel **(A)**. Data are expressed as percentage of cytokine release relative to control medium. Mean values ± SD are shown, *n* = 6. **p < 0.01.

Based on our findings and data from the literature showing an increased number of activated Vδ2 T cells in intestinal mucosa from CD patients (27), we sought to investigate whether TLR/PRR activating structures likely present in inflamed intestinal microenvironment could affect the activation potential of γδ T cells. To this aim, blood γδ T lymphocytes from healthy donors were stimulated with IPP in the presence of CM of IEC, isolated from colonic biopsies of either subjects with endoscopically active CD or sex/age matched HC. As shown in Figure 8A, IPP-induced activation, as assessed by IFNγ production, was significantly enhanced when γδ T lymphocytes were exposed to CM of IEC from active CD patients as compared to control medium. Conversely, CM of IEC from HC did not significantly affect IFNγ release (Figure 8A). Interestingly, a significant reduction in CD CM-induced γδ T cell activation was observed when these cells were stimulated with IPP in the presence of TLR7/8/9 targeting ODN (Figure 8B), thus suggesting the presence of TLR targeting T cell co-activating structures in the microenvironment of patient inflamed epithelium.

## Discussion

The TLR7/8 agonist R848 has been extensively studied for its adjuvant activity and its effects on immune responses following either topic or oral administration. The main adjuvant activity has been so far attributed to its capacity to induce the production of immune mediators and the activation of DC, thereby leading to the enhancement of both humoral and cellular immune responses. Moreover, data from the literature suggest that most of the responses induced by oral or topic R848 delivery likely result from agonist diffusion within tissues and in the circulation thus leading to direct immune cell stimulation. The results of this study not only expand the range of cellular targets and immune responses influenced by R848 but also provide novel evidence that some of these responses might require the cooperation of agonist-conditioned tissues, such as the intestinal epithelium, thus highlighting the importance of local microenvironment in shaping immune responses.

Specifically, we demonstrate herein that R848 is easily transported across the Caco-2 cell-derived polarized IEC monolayer, showing permeability coefficients typical of completely adsorbed drugs. Consistent with our finding, previous studies performed in mice orally injected with the agonist had suggested that this molecule could easily cross the gut mucosa (14, 15). Furthermore, it was reported that, following intracolonic administration of R848 in mice, a systemic inflammatory response was induced in the absence of epithelium disruption, consistent with a rapid penetration of R848 across the intestinal wall into the draining lymphatic system (28). As a further evidence, topical R848 application was found to confer robust resistance to mice against intravenous challenge with virulent *Leishmania* strains, in keeping with a rapid and efficient transfer of the agonist into the underlying tissues and into the circulation, responsible for systemic immunization (29).

Our results also unravel the effects of TLR8 stimulation of IEC on the phenotype/function of innate immunity cells. Specifically, we report for the first time that apical exposure of polarized Caco-2 cell monolayer to R848 drives the enrichment of the inflammatory CD14^+^CD16^+^ monocyte subpopulation by inducing the production of CCL2 by epithelial cells. In keeping with this finding, data previously obtained *in vivo* in non-human primates (25) highlighted that R848 injection leads to a decrease of myeloid DC number in the blood and a concomitant enrichment of CD14^+^CD16^+^ cells, likely due to the induction of pro-inflammatory cytokines/chemokines. Interestingly, it has been shown that *in vitro* exposure of human monocytes to CCL2 results in the enrichment of the CD14^+^CD16^+^ cell subset and that this chemokine is responsible for inflammatory monocyte accumulation induced by breast cancer cell microenvironment (30). Accordingly, we demonstrate that R848-induced CCL2 in polarized Caco-2 cell monolayer strongly contributes to the accumulation of CD14/CD16 double positive cells, whereas no effect is exerted by IL-6 or type I IFN. As a further evidence that the induction of this cell population is dependent on IEC response to R848, we show that it does not occur following direct exposure of monocytes to the agonist. In this regard, although R848 was shown to regulate the expression and function of different Fcγ receptor members, with both activating and inhibitory functions, no direct effect was reported on the expression of CD16/FcγRIII (31). The enrichment of the CD14^+^CD16^+^ cell population, previously associated with chronic inflammatory conditions (32), at mucosa level might contribute to the strong inflammatory reactions that characterize R848 activity in exposed animals.

We also report that R848, either transported through the IEC monolayer or directly administered to freshly isolated γδ T cells, enhances the phosphoantigen-induced activation of these innate lymphocytes. Specifically, significantly higher levels of IFNγ are produced when the γδ TCR is triggered in the presence of R848. Although it has been shown that human γδ T lymphocytes express different TLR, including TLR7 and TLR8, to the best of our knowledge this is the first demonstration that TLR7/8 ligands can potentiate their activation. Furthermore, the selective requirement of TLR8 for this effect was also shown. Additionally, our results indicate that, at tissue level, the mechanisms of γδ T cell activation can be more complex and finely regulated. In fact we show that, although R848 transported across the IEC monolayer exerts a promoting effect on phosphoantigen-induced direct activation of γδ T lymphocytes, it does not counteract the negative control exerted by IEC microenvironment on the DC-dependent activation of these cells. This suggests that tissue microenvironment may regulate the balance between activating and inhibitory factors and ultimately dictate the type of response. In keeping with this hypothesis, and with results from other groups (33, 34), we report that this compound exerts immunosuppressive activities such as the inhibition of DC differentiation and of Th1 type response generation.

Overall, the enhancing effect of R848 on γδ T lymphocyte activation would be particularly relevant considering the key role of these cells in the first-line defense against infections and tumors (35). Notably, these unconventional T cells represent attractive effectors for cancer immunotherapy (36). On the other hand, the generation of DC characterized by a reduced capacity to induce both conventional and γδ T lymphocyte-mediated responses, would limit the inflammatory response and/or contribute to immunosuppression. The opposite, pro-inflammatory vs. regulatory, effects are consistent with the dual role of TLR7/8 agonists in driving either protective immunity or chronic inflammation/autoimmunity (3, 37). The induction of anti-inflammatory/regulatory pathways (IL-10 production and Treg cell expansion) has also been reported to limit the adjuvant activity of other TLR agonists (38). These findings highlight the complexity of effects that can be elicited by TLR agonists, depending on the target cell type, and the importance of the administration route in regulating the intensity of response and in balancing apparently opposite effects.

In addition to their relevance in the mechanism of action of R848, our data argue for an involvement of γδ T and CD14^+^CD16^+^ cell-mediated responses during natural viral infections, when TLR8 ligands are produced. In particular, expansion of these cell populations has been described in some viral infections (32, 39, 40) where they play either protective or pathogenic roles, as well as in chronic inflammatory conditions characterized by break of gut homeostasis and microbial translocation (27, 41). Notably, we show that the CM of IEC isolated from subjects with active CD strongly enhances the activation of phosphoantigen responsive control γδ T lymphocytes with respect to IEC from healthy donors. Accordingly, it has been recently reported that an increased number of activated Vδ2 T cells is detected in intestinal mucosa from CD patients (27). Although different mechanisms can be responsible for this effect, we demonstrate that TLR-activating structures released at the level of inflamed epithelium strongly contribute to γδ T lymphocyte activation induced by intestinal microenvironment of active CD patients. The response of epithelium itself to TLR stimulation might in turn potentiate, through the release of pro-inflammatory mediators, the effects of microbial products on immune cells thus enhancing/perpetuating inflammation. In this regard, TLR8 expression has been reported to be selectively activated in inflamed colonic epithelium of IBD-affected subjects (6, 7) and its mucosal expression directly correlates with the severity of intestinal inflammation (8).

Altogether, the results of this study increase our knowledge on the molecular mechanisms and cellular responses that might be triggered by TLR8 stimulation. They also add novel information on the mechanism(s) through which R848 exerts its adjuvant activity as well as on the role that the delivery route can play in regulating TLR agonist-induced responses. A better knowledge of the mechanisms and pathways regulated through TLR stimulation as well as of the role of TLR agonist administration route in shaping immune responses might contribute to the design of more effective strategies for microbial and cancer vaccines, as well as to better understand the role of microbial molecular patterns in infectious diseases and in chronic inflammation.

## Ethics Statement

This study has been conducted in accordance with the Declaration of Helsinki and, according to national and international guidelines, it was approved by the review board of Istituto Superiore di Sanità (project identification code: CE/11/299). All the subjects included were provided with complete information about the study and asked to sign an informed consent.

## Author Contributions

CA designed and performed the experiments; BV performed the experiments; MF performed the biochemical analyses; PP analyzed the data and provided intellectual input; AB and AM performed TLR gene expression profiles; SG provided intellectual input throughout the study; LC conceived the study, supervised work, analyzed data, and wrote the manuscript.

## Conflict of Interest Statement

The research was conducted in the absence of any commercial or financial relationships that could be construed as a potential conflict of interest.

## Abbreviations

AS, apical side; BS, basolateral side; CD, Crohn’s disease; CDEIS, Crohn’s Disease Endoscopic Index of Severity; CM, conditioned medium; DC, dendritic cell; IBD, inflammatory bowel disease; IEC, intestinal epithelial cells; IPP, isopentenilpyrophosphate; ODN, oligonucleotides; PRR, pattern recognition receptor; TEER, transepithelial electrical resistance; TLR, toll-like receptor; TM, transport medium; ZOL, zoledronate.

## References

[1] Kawai T Akira S. The role of pattern-recognition receptors in innate immunity: update on toll-like receptors. Nat Immunol (2010) 11(5):373–84. 10.1038/ni.1863 20404851

[2] Ohto U Tanji H Shimizu T Structure and function of toll-like receptor 8. Microbes Infect (2014) 16(4):273–82. 10.1016/j.micinf.2014.01.007 24513445

[3] Diebold SS Recognition of viral single-stranded RNA by toll-like receptors. Adv Drug Deliv Rev (2008) 60(7):813–23. 10.1016/j.addr.2007.11.004 18241955

[4] Grasa L Abecia L Forcen R Castro M de Jalon JA Latorre E Antibiotic-induced depletion of murine microbiota induces mild inflammation and changes in toll-like receptor patterns and intestinal motility. Microb Ecol (2015) 70(3):835–48. 10.1007/s00248-015-0613-8 25896428

[5] Fukata M Arditi M. The role of pattern recognition receptors in intestinal inflammation. Mucosal Immunol (2013) 6(3):451–63. 10.1038/mi.2013.13 23515136 PMC3730813

[6] Steenholdt C Andresen L Pedersen G Hansen A Brynskov J. Expression and function of toll-like receptor 8 and Tollip in colonic epithelial cells from patients with inflammatory bowel disease. Scand J Gastroenterol (2009) 44(2):195–204. 10.1080/00365520802495529 18985539

[7] Abreu MT. Toll-like receptor signalling in the intestinal epithelium: how bacterial recognition shapes intestinal function. Nat Rev Immunol (2010) 10(2):131–44. 10.1038/nri2707 20098461

[8] Sanchez-Munoz F Fonseca-Camarillo G Villeda-Ramirez MA Miranda-Perez E Mendivil EJ Barreto-Zuniga R Transcript levels of toll-like receptors 5, 8 and 9 correlate with inflammatory activity in Ulcerative Colitis. BMC Gastroenterol (2011) 11:138. 10.1186/1471-230X-11-138 22185629 PMC3287145

[9] Helminen O Huhta H Lehenkari PP Saarnio J Karttunen TJ Kauppila JH. Nucleic acid-sensing toll-like receptors 3, 7 and 8 in esophageal epithelium, Barrett’s esophagus, dysplasia and adenocarcinoma. Oncoimmunology (2016) 5(5):e1127495. 10.1080/2162402X.2015.1127495 27467941 PMC4910747

[10] Hemmi H Kaisho T Takeuchi O Sato S Sanjo H Hoshino K Small anti-viral compounds activate immune cells via the TLR7 MyD88-dependent signaling pathway. Nat Immunol (2002) 3(2):196–200. 10.1038/ni758 11812998

[11] Ahonen CL Gibson SJ Smith RM Pederson LK Lindh JM Tomai MA Dendritic cell maturation and subsequent enhanced T-cell stimulation induced with the novel synthetic immune response modifier R-848. Cell Immunol (1999) 197(1):62–72. 10.1006/cimm.1999.1555 10555997

[12] Wagner TL Ahonen CL Couture AM Gibson SJ Miller RL Smith RM Modulation of TH1 and TH2 cytokine production with the immune response modifiers, R-848 and imiquimod. Cell Immunol (1999) 191(1):10–9. 10.1006/cimm.1998.1406 9918682

[13] Vasilakos JP Smith RM Gibson SJ Lindh JM Pederson LK Reiter MJ Adjuvant activities of immune response modifier R-848: comparison with CpG ODN. Cell Immunol (2000) 204(1):64–74. 10.1006/cimm.2000.1689 11006019

[14] Yrlid U Cerovic V Milling S Jenkins CD Klavinskis LS MacPherson GG A distinct subset of intestinal dendritic cells responds selectively to oral TLR7/8 stimulation. Eur J Immunol (2006) 36(10):2639–48. 10.1002/eji.200636426 16983724

[15] Yrlid U Milling SW Miller JL Cartland S Jenkins CD MacPherson GG Regulation of intestinal dendritic cell migration and activation by plasmacytoid dendritic cells, TNF-alpha and type 1 IFNs after feeding a TLR7/8 ligand. J Immunol (2006) 176(9):5205–12. 10.4049/jimmunol.176.9.5205 16621985

[16] Vasilakos JP Tomai MA. The use of toll-like receptor 7/8 agonists as vaccine adjuvants. Expert Rev Vaccines (2013) 12(7):809–19. 10.1586/14760584.2013.811208 23885825

[17] Zhang J Lu Y Wei J Li L Han L Protective effect of carboxytmethylpachymaran on TNF-alpha-induced damage in Caco-2 cell monolayers. Int J Biol Macromol (2016) 93(Pt A):506–11. 10.1016/j.ijbiomac.2016.07.095 27477245

[18] Gupta V Doshi N Mitragotri S. Permeation of insulin, calcitonin and exenatide across Caco-2 monolayers: measurement using a rapid, 3-day system. PLoS One (2013) 8(2):e57136. 10.1371/journal.pone.0057136 23483881 PMC3586668

[19] Conti L Casetti R Cardone M Varano B Martino A Belardelli F Reciprocal activating interaction between dendritic cells and pamidronate-stimulated gammadelta T cells: role of CD86 and inflammatory cytokines. J Immunol (2005) 174(1):252–60. 10.4049/jimmunol.174.1.252 15611247

[20] Mary JY Modigliani R Development and validation of an endoscopic index of the severity for Crohn’s disease: a prospective multicentre study. Groupe d’Etudes Therapeutiques des Affections Inflammatoires du Tube Digestif (GETAID). Gut (1989) 30(7):983–9. 10.1136/gut.30.7.983 2668130 PMC1434265

[21] Seidelin JB Horn T Nielsen OH. Simple and efficient method for isolation and cultivation of endoscopically obtained human colonocytes. Am J Physiol Gastrointest Liver Physiol (2003) 285(6):G1122–8. 10.1152/ajpgi.00533.2002 14613919

[22] Artursson P Palm K Luthman K. Caco-2 monolayers in experimental and theoretical predictions of drug transport. Adv Drug Deliv Rev (2001) 46(1–3):27–43. 10.1016/S0169-409X(00)00128-9 11259831

[23] Nisini R Torosantucci A Romagnoli G Chiani P Donati S Gagliardi MC beta-Glucan of *Candida albicans* cell wall causes the subversion of human monocyte differentiation into dendritic cells. J Leukoc Biol (2007) 82(5):1136–42. 10.1189/jlb.0307160 17656653

[24] Ziegler-Heitbrock L. The CD14+ CD16+ blood monocytes: their role in infection and inflammation. J Leukoc Biol (2007) 81(3):584–92. 10.1189/jlb.0806510 17135573

[25] Kwissa M Nakaya HI Oluoch H Pulendran B. Distinct TLR adjuvants differentially stimulate systemic and local innate immune responses in nonhuman primates. Blood (2012) 119(9):2044–55. 10.1182/blood-2011-10-388579 22246032 PMC3311246

[26] Pietschmann K Beetz S Welte S Martens I Gruen J Oberg HH Toll-like receptor expression and function in subsets of human gammadelta T lymphocytes. Scand J Immunol (2009) 70(3):245–55. 10.1111/j.1365-3083.2009.02290.x 19703014

[27] McCarthy NE Hedin CR Sanders TJ Amon P Hoti I Ayada I Azathioprine therapy selectively ablates human Vdelta2(+) T cells in Crohn’s disease. J Clin Invest (2015) 125(8):3215–25. 10.1172/JCI80840 26168223 PMC4563752

[28] Karlsson A Jagervall K Utkovic H Karlsson L Rehnstrom E Fredin MF Intra-colonic administration of the TLR7 agonist R-848 induces an acute local and systemic inflammation in mice. Biochem Biophys Res Commun (2008) 367(2):242–8. 10.1016/j.bbrc.2007.12.046 18083110

[29] Craft N Birnbaum R Quanquin N Erfe MC Quant C Haskell J Topical resiquimod protects against visceral infection with *Leishmania infantum* chagasi in mice. Clin Vaccine Immunol (2014) 21(9):1314–22. 10.1128/CVI.00338-14 25030052 PMC4178562

[30] Feng AL Zhu JK Sun JT Yang MX Neckenig MR Wang XW CD16+ monocytes in breast cancer patients: expanded by monocyte chemoattractant protein-1 and may be useful for early diagnosis. Clin Exp Immunol (2011) 164(1):57–65. 10.1111/j.1365-2249.2011.04321.x 21361908 PMC3074217

[31] Butchar JP Mehta P Justiniano SE Guenterberg KD Kondadasula SV Mo X Reciprocal regulation of activating and inhibitory Fc{gamma} receptors by TLR7/8 activation: implications for tumor immunotherapy. Clin Cancer Res (2010) 16(7):2065–75. 10.1158/1078-0432.CCR-09-2591 20332325 PMC2848878

[32] Wong KL Yeap WH Tai JJ Ong SM Dang TM Wong SC. The three human monocyte subsets: implications for health and disease. Immunol Res (2012) 53(1–3):41–57. 10.1007/s12026-012-8297-3 22430559

[33] Assier E Marin-Esteban V Haziot A Maggi E Charron D Mooney N. TLR7/8 agonists impair monocyte-derived dendritic cell differentiation and maturation. J Leukoc Biol (2007) 81(1):221–8. 10.1189/jlb.0705385 17023556

[34] Hackstein H Knoche A Nockher A Poeling J Kubin T Jurk M The TLR7/8 ligand resiquimod targets monocyte-derived dendritic cell differentiation via TLR8 and augments functional dendritic cell generation. Cell Immunol (2011) 271(2):401–12. 10.1016/j.cellimm.2011.08.008 21889130

[35] Poggi A Zocchi MR gammadelta T lymphocytes as a first line of immune defense: old and new ways of antigen recognition and implications for cancer immunotherapy. Front Immunol (2014) 5:575 10.3389/fimmu.2014.00575 25426121 PMC4226920

[36] Hannani D Ma Y Yamazaki T Dechanet-Merville J Kroemer G Zitvogel L Harnessing gammadelta T cells in anticancer immunotherapy. Trends Immunol (2012) 33(5):199–206. 10.1016/j.it.2012.01.006 22364810

[37] Yokogawa M Takaishi M Nakajima K Kamijima R Fujimoto C Kataoka S Epicutaneous application of toll-like receptor 7 agonists leads to systemic autoimmunity in wild-type mice: a new model of systemic *Lupus erythematosus*. Arthritis Rheumatol (2014) 66(3):694–706. 10.1002/art.38298 24574230

[38] Engel AL Holt GE Lu H. The pharmacokinetics of toll-like receptor agonists and the impact on the immune system. Expert Rev Clin Pharmacol (2011) 4(2):275–89. 10.1586/ecp.11.5 21643519 PMC3105468

[39] Latha TS Reddy MC Durbaka PV Rachamallu A Pallu R Lomada D gammadelta T cell-mediated immune responses in disease and therapy. Front Immunol (2014) 5:571 10.3389/fimmu.2014.00571 25426120 PMC4225745

[40] Ahout IM Jans J Haroutiounian L Simonetti ER van der Gaast-de Jongh C Diavatopoulos DA Reduced expression of HLA-DR on monocytes during severe respiratory syncytial virus infections. Pediatr Infect Dis J (2016) 35(3):e89–96. 10.1097/INF.0000000000001007 26658377

[41] Koch S Kucharzik T Heidemann J Nusrat A Luegering A. Investigating the role of proinflammatory CD16+ monocytes in the pathogenesis of inflammatory bowel disease. Clin Exp Immunol (2010) 161(2):332–41. 10.1111/j.1365-2249.2010.04177.x 20456413 PMC2909416

