# Supplementary figures and images for: Direct and Intestinal Epithelial Cell-Mediated Effects of TLR8 Triggering on Human Dendritic Cells, CD14+CD16+ Monocytes and γδ T Lymphocytes
Source: Front Immunol. 2017 Dec 22;8:1813. doi: 10.3389/fimmu.2017.01813 (PMC5743793; doi:10.3389/fimmu.2017.01813)

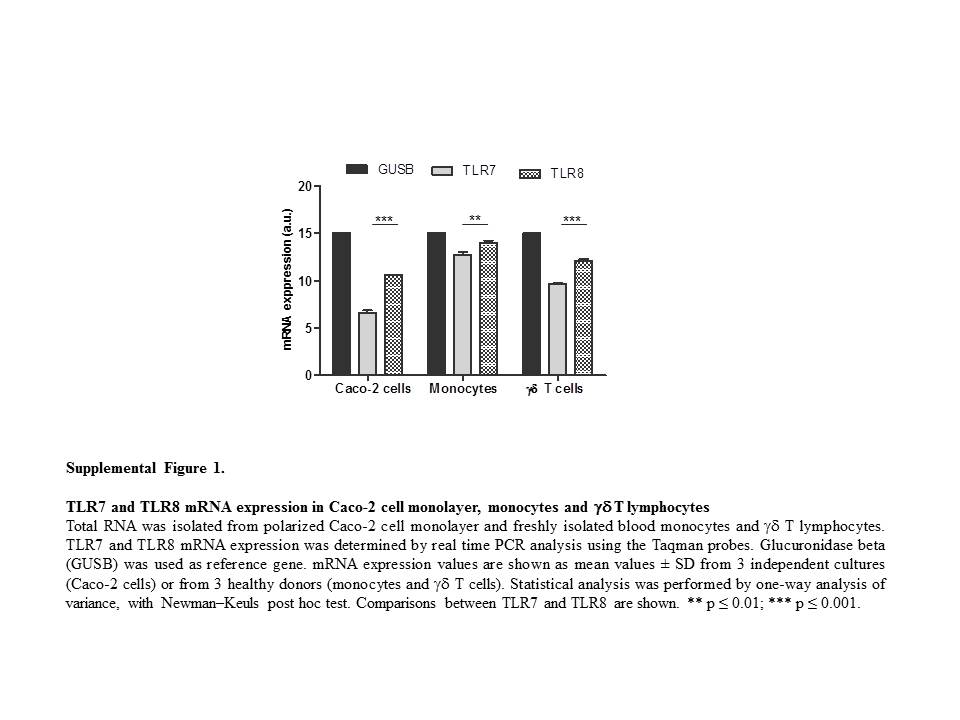

Supplement: Supplementary file 1 [file Image_1.JPEG]
